# The emergence of neurodiplomacy

**DOI:** 10.1016/j.isci.2022.104370

**Published:** 2022-05-17

**Authors:** Mohammed A. Mostajo-Radji

**Affiliations:** 1 UCSC Genomics Institute, University of California Santa Cruz, Santa Cruz, CA 95060, USA

## Abstract

Nearly 1 in 6 people suffer from neurological disorders. As large multinational and interdisciplinary scientific collaborations in neuroscience emerge, how do we ensure equitable voice in the development and access to these technologies? In this backstory, neurodiplomacy is proposed as a new field to advance the Sustainable Development Goals and scientific discovery through cooperation between governments, academics, nonprofit organizations, and entrepreneurs.

**Figure:**
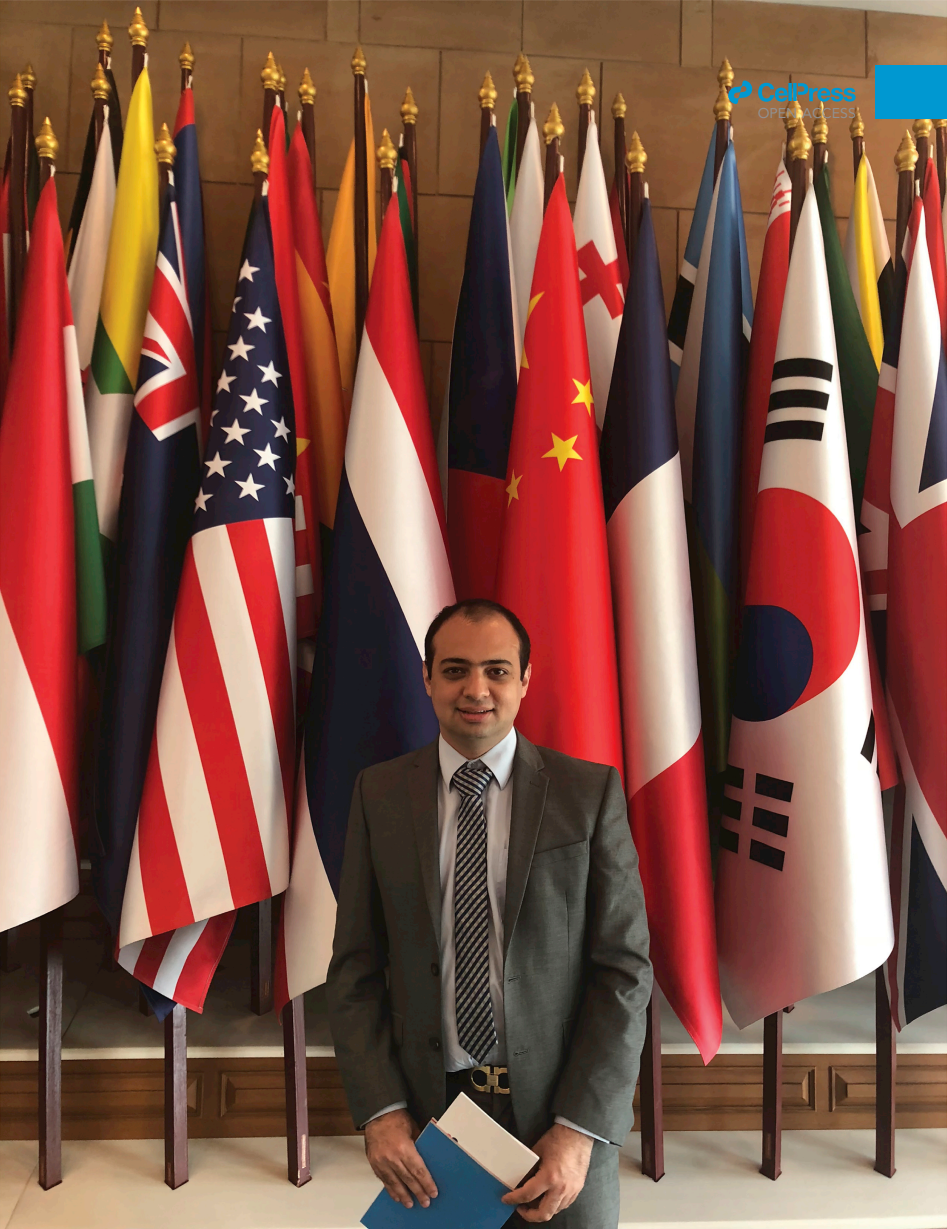
Neurodiplomacy is expanding in the Global South Mostajo-Radji as part of the South American delegation to the Kingdom of Thailand in 2018.

‘’ There is little overlap in training between disciplines: policymakers and diplomats are not usually trained in the core sciences. At the same time, scientists have no training in international affairs and global issues. So, it is very hard to understand each other when they are at the same table.’’

‘’ I have focused on science education as a non-state form of diplomacy in Latin America. To this end, context dependent education is key to success’’

‘’ In the eyes of the public, particularly in Latin America, it is hard to separate scientific advice to governments and science diplomacy from politics’’

‘’ I believe the field will focus in five areas: neurorights, data governance, trade of neurotechnologies, education, and people-to-people exchange.’’

In the global arena, communities are represented by diplomatic missions in other countries and multinational organizations. Often, multinational interests take the form of complex science, technology, and innovation (STI) issues, requiring the formation of interdisciplinary teams including scientists, engineers, policy advisors, and diplomats. The field of science diplomacy was born at the intersection between STI and international relations to understand, advise, and advance these collaborations.

In this Backstory, Dr. Mohammed Mostajo-Radji brings attention to Neurodiplomacy as an emerging branch of science diplomacy to study international collaborations regarding neuroscience, neurotechnologies, and artificial intelligence. Dr. Mostajo-Radji is an Assistant Research Scientist at the University of California Santa Cruz and part of the Brainengineers group (https://braingeneers.ucsc.edu/). He leads an interdisciplinary team studying neuronal interactions during cortical development and creating Internet of Things-enabled technologies for experimental science education.

## Main text

### Proximity

What has led to the emergence of neurodiplomacy? Why do we need a cross-talk between governments, nonprofit organizations, academics, entrepreneurs, and the general public?

Humans have always been fascinated by the brain. Even if you look at medical illustrations of ancient Greece, you will find attempts to map the brain. This fascination continues today. With the development of neurotechnologies and big data, new questions have emerged to tackle issues such as data governance of neuronal data and the representation of diverse groups in neuroscience discoveries. The reality is that to date, there is still a big disconnection between governments and academia, entrepreneurship, and nonprofit organizations performing work in neuroscience. This gap has started to close over the past decade with projects such as the BRAIN Initiative (Insel et al., 2013). Yet, there is a lot of work to be done to ensure equity and inclusion, both in the scientific discovery as well as in the access to the technologies developed (Barber and Mostajo-Radji, 2020).

What is the role of Latin America in neurodiplomacy?

I believe Latin America is becoming an unexpected leader in science diplomacy and neurodiplomacy. One can look at some examples in Latin America that are pretty remarkable. The opening ceremony of the 2014 World Cup in Sao Paulo highlighted, for the first time, scientific achievement in a global sports event: the initial kick of the event was given by a paraplegic man wearing an exoskeleton designed by Miguel Nicolelis and his team of over 150 scientists and engineers from around the world. The story behind this achievement is an outstanding example of government-academia partnerships that was funded not only by Brazil but also by governments abroad. Therefore, it should not be a surprise that the World Congress of the International Brain Research Organization (IBRO) was hosted in Brazil a year later, marking the first time that a global neuroscience event took place in Latin America. However, I would argue that the catapulting moment for Latin America in neurodiplomacy came during the Zika outbreak of 2015–2016, where research groups from the region, as well as the developed world, had to come together to generate data, share information and find solutions to the disease. To my knowledge, this was the first time in which multinational organizations, such as the European Union, funded large neuroscience-focused projects in the region.

At the diplomatic level, Latin America has made major advances in neurodiplomacy. I am honored to have been the first formal STI ambassador of a Latin American country in 2020. However, the region has been actively working in this field for a long time. In 2014, for example, Costa Rica appointed Roman Macaya-Hayes, a UCLA-trained biochemist, as its ambassador to the United States. Several other Latin American countries have delegated their STI interests to their consulates or embassies: the consulates of Uruguay, Mexico, and Colombia, for instance, have made major advances in STI collaborations through their consulates in Boston and San Francisco.

**Figure:**
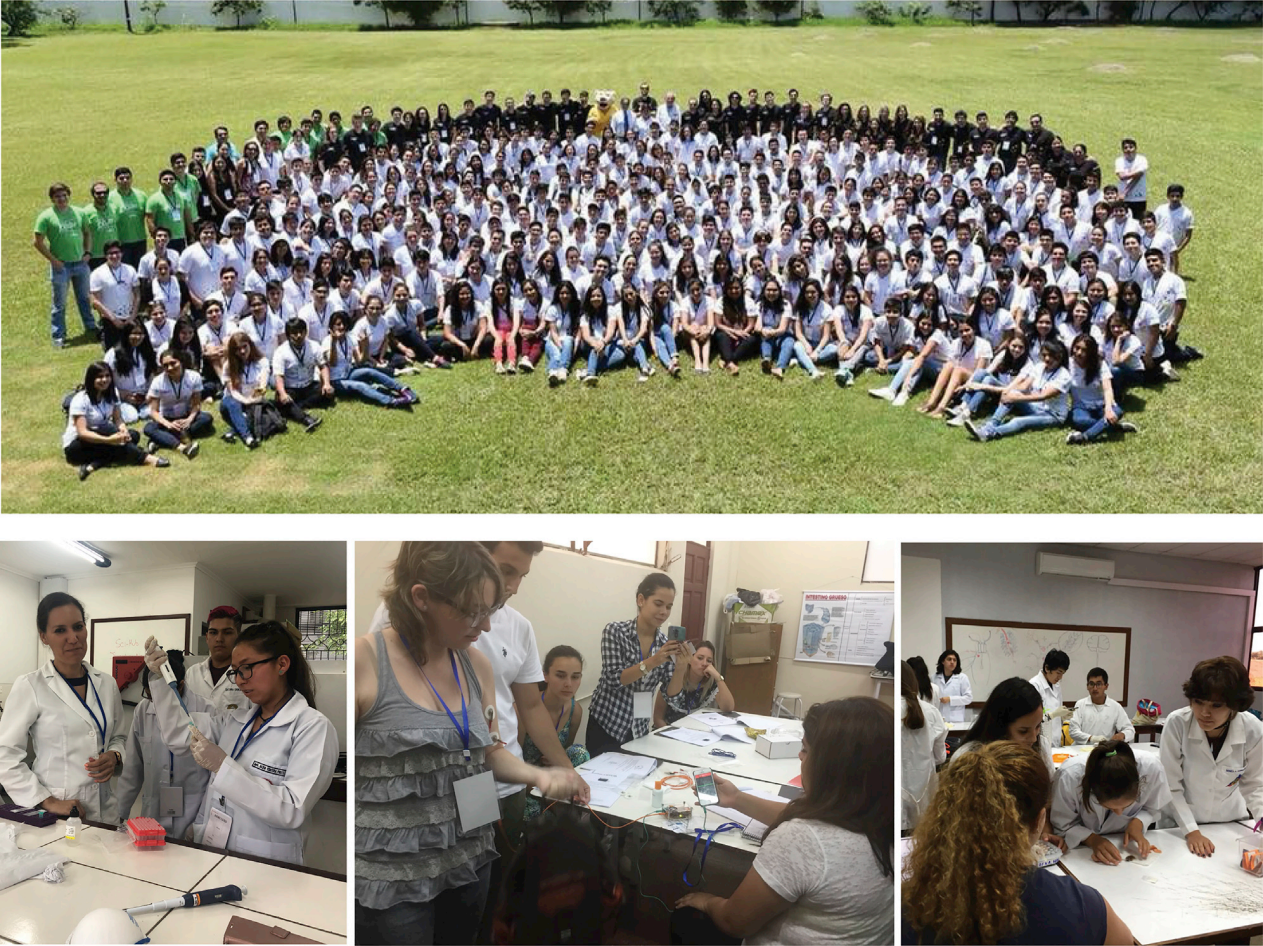
Science education as a nonstate approach of science diplomacy Clubes de Ciencia Bolivia, a program sponsored by the US Department of State, trained thousands of students in scientific topics. The most seeked courses were neuroscience, genome engineering, and scientific entrepreneurship (Ferreira et al., 2019).

What are the opportunities for cooperation, and how do individual scientists/groups fit into the field’s interdisciplinary nature?

I genuinely believe that we are close to a revolution in neuroscience. But if history has taught us something, it is that this revolution can only occur if we start involving players from many fields and many regions. In genomics, we have moved from the human genome to the human pangenome as the next frontier to incorporate genetic diversity in the area. It has become clear that a higher diversity of human-induced pluripotent stem cell lines are required to make applicable and scalable discoveries in the developmental biology field. As more mega projects in neuroscience appear, we need to start thinking about involving this diversity from the very beginning. Here is where I see an important role for laboratories worldwide to come together as part of these projects.

### Language

What are the challenges of multinational collaborations and communication between neuroscientists, social scientists, and policymakers? How can we resolve them?

I would say there are five major challenges that we need to overcome, most of them being common with other branches of science diplomacy:

**Figure:**
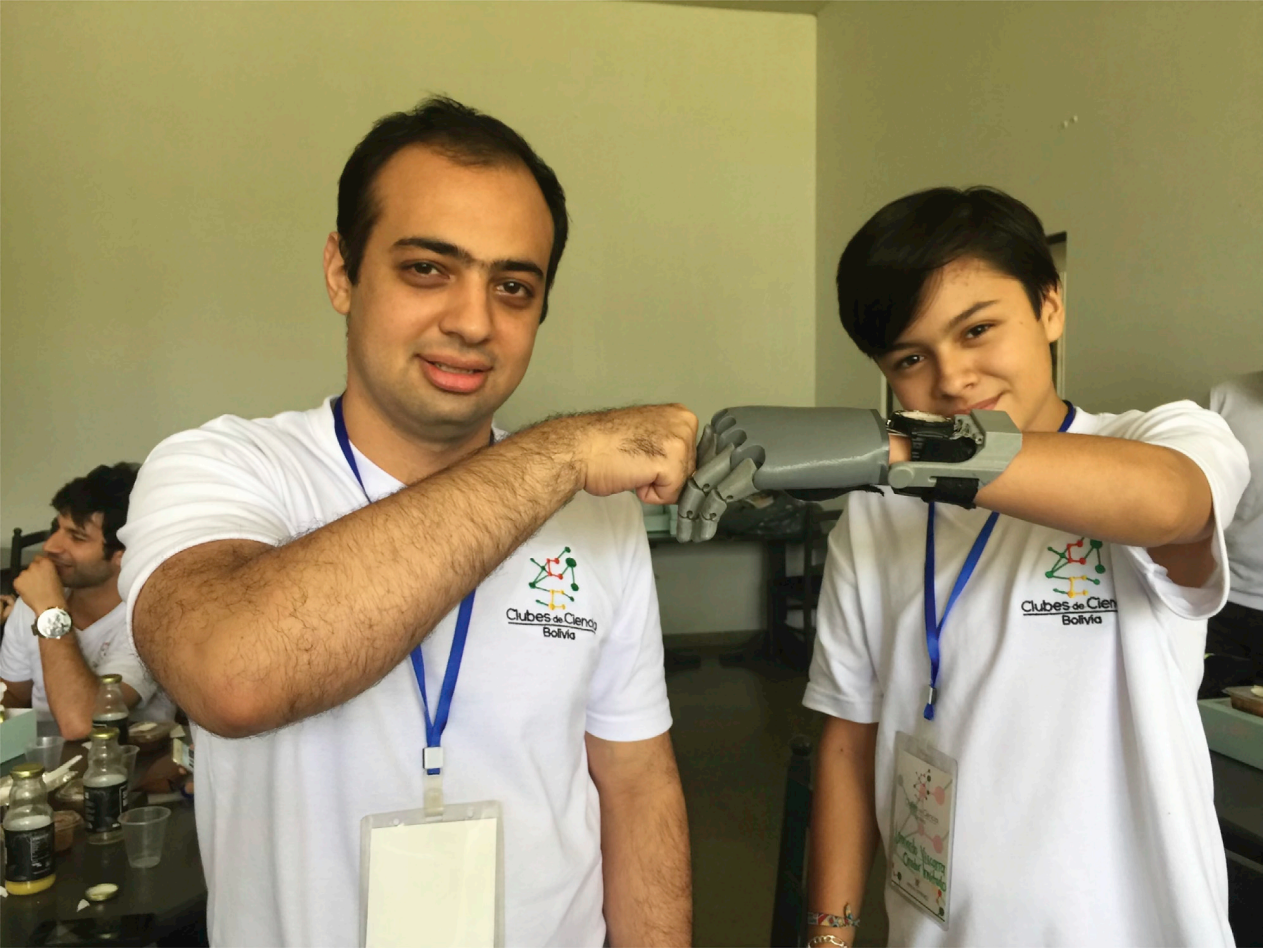
Science education can have long-lasting effects in society Mostajo-Radji with a student in a 3D printing of prosthetics course taught through a collaboration with the US Embassy in Bolivia. The student, who has been missing a limb since birth, continued to create a nonprofit organization that provides free low-cost 3D printed prosthetics to underprivileged children in Bolivia.

1) There is little overlap in training between disciplines: policymakers and diplomats are not usually trained in the core sciences. At the same time, scientists have no training in international affairs and global issues. So, it is very hard to understand each other when they are at the same table. We need to start having people ask themselves questions that are interdisciplinary in nature. For example, scientists rarely think about how their specific work contributes to initiatives like the United Nations Sustainable Development Goals (SDGs). Similarly, diplomats and policymakers are reluctant to ask themselves who are the world experts on a particular topic they will discuss that day and how to reach out to them to get advice.
2) We do not train scientists to be good communicators. As such, many scientists lack the understanding of how to communicate with the public and with experts in other fields. In a way, academia has failed to recognize the importance of science communication and outreach, as these components are not evaluated and are not considered relevant to advance your scientific career.

**Figure:**
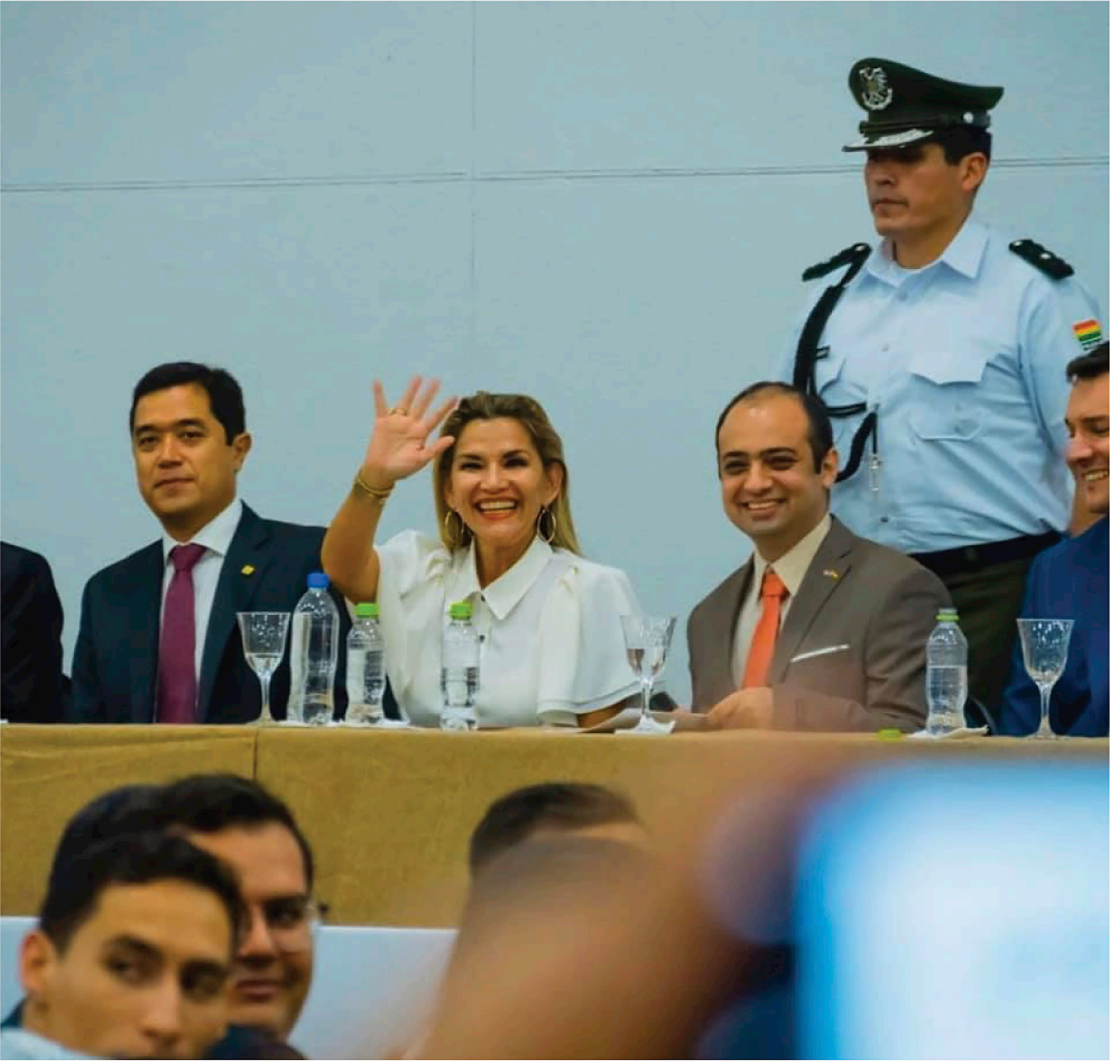
Science diplomacy brings together education, government, and industry President Jeanine Añez, members of the US Embassy in Bolivia, the Bolivian Chamber of Industry and Commerce, and Mostajo-Radji at Clubes de Ciencia Bolivia.
3) Historically, many countries in the Global South have experienced "helicopter science": scientists coming from the developed world, taking samples, publishing papers, and going away. This phenomenon has created a feeling of mistrust, which can only be overcome by making everyone an equal partner.
4) As per sample acquisition, we still have a very North-focused approach when it comes to informed consent. Most international projects have a single type of informed consent form that gets directly translated into the languages of all participating countries. This is problematic because it disregards cultural contexts that may influence the participants' understanding. In my experience, patients in the Global North tend to be very protective about sharing their personal biological data. In the Global South, I see a lot more willingness of patients to share their data. This stems from the fact that people in the Global South are usually very grateful that someone is listening to them and feel compelled to share their samples to reciprocate. Several groups have gone around this dilemma by reducing the number of sample sources and assuming that one group can be representative of others. For example, we often study Latinx living in the United States and assume they represent the Latin American population. However, at least for neuroscience and mental illness, the data show us that this assumption is not true (Alegria et al., 2008). As more mega projects arise, we need to start thinking about ways to "destandardize" these forms.

**Figure:**
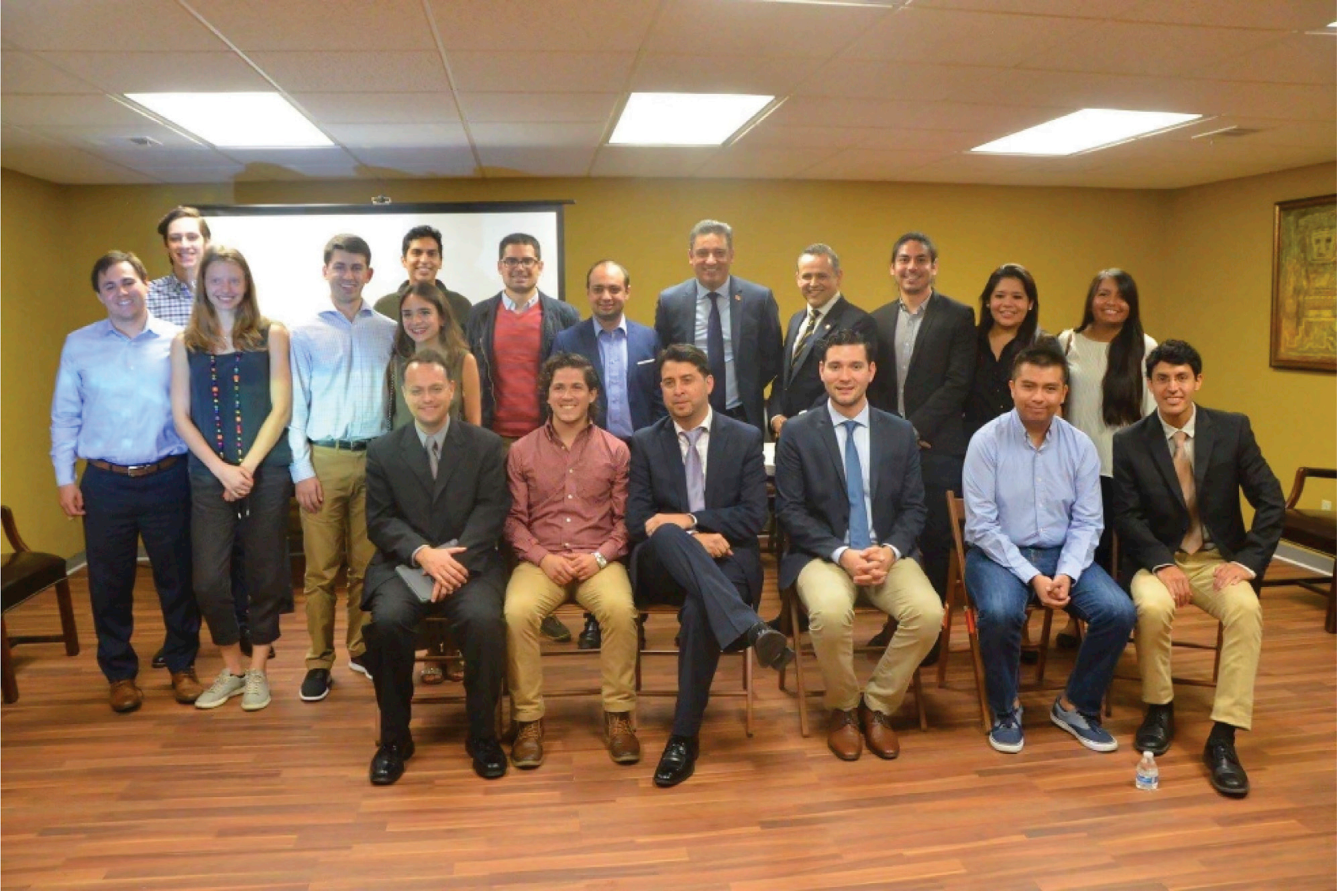
Science diplomacy also involves South-North collaborations Through a collaboration with the then Chargé d’affaires of Bolivia to the United States, Mr. Pablo Canedo, Mostajo-Radji and his team created several courses for American children of Bolivian immigrants that were taught by Bolivian instructors at the Bolivian Embassy in Washington DC.
5) Neuroscience data is intrinsically multimodal: from imaging and connectomics to RNA sequencing and electric data. So even before we get to policy advisors and diplomats, there is still quite a bit of work to be done integrating these data types in a coherent and permissible way that could then be summarized in succinct policy briefs. The California Institute for Regenerative Medicine and the US National Institutes of Health are taking the lead on this front. However, I think there will be a lot of work to be done in data integration, particularly when multiple countries with different regulations generate the data.

### Research methods

Describe your approach to developing or adjust the methodology for advancing neurotechnology and neurodiplomacy (especially in the context of Latin America).

Personally, I have focused on science education as a non-state form of diplomacy in Latin America. To this end, context dependent education is key to success (Carosso et al., 2019a). A few years ago, we did a large survey involving high school and college students where we asked them what the main topics of interest to them in STI are. To our surprise, neurotechnologies and genome engineering were consistently ranked as the top areas of interest among students (Ferreira et al., 2019). Integrating feedback from the local communities and knowledge from local scientists, we have shown that we can advance diplomatic relations through education and scientific collaborations (Carosso et al., 2019b).

**Figure:**
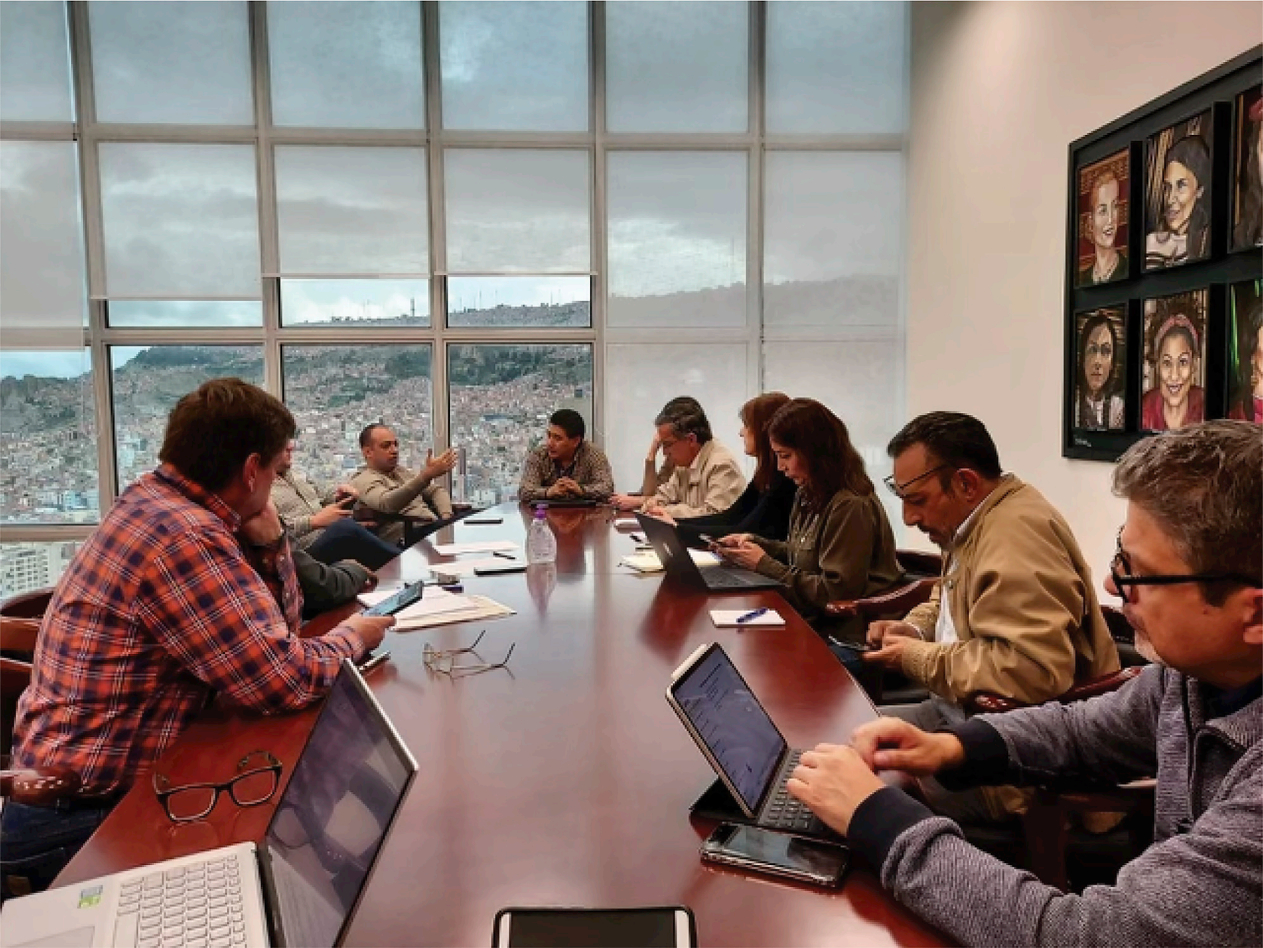
The COVID-19 pandemic required high-level science diplomacy conversations Mostajo-Radji, along with other scientists, in discussions with representatives of the World Health Organization and the United Nations Development Program in La Paz during early days of the COVID-19 pandemic.

What are the main challenges you faced so far in your career and research?

In the eyes of the public, particularly in Latin America, it is hard to separate scientific advice to governments and science diplomacy from politics. We are often put in the middle of a storm for disagreements from an opposition that is acting with a political agenda instead of looking at the global picture. For example, one can look at the COVID-19 response in Latin America, where people decided to use chlorine dioxide—a type of bleach—to treat the disease. Although no government-approved this chemical, many countries had politicians pushing for its use (Mostajo-Radji, 2021). I was particularly shocked by the statement of a Peruvian congressman who claimed, "You didn’t need to be a scientist to know it works," unveiling precisely my point that these disagreements had no scientific reasoning behind them.

**Figure:**
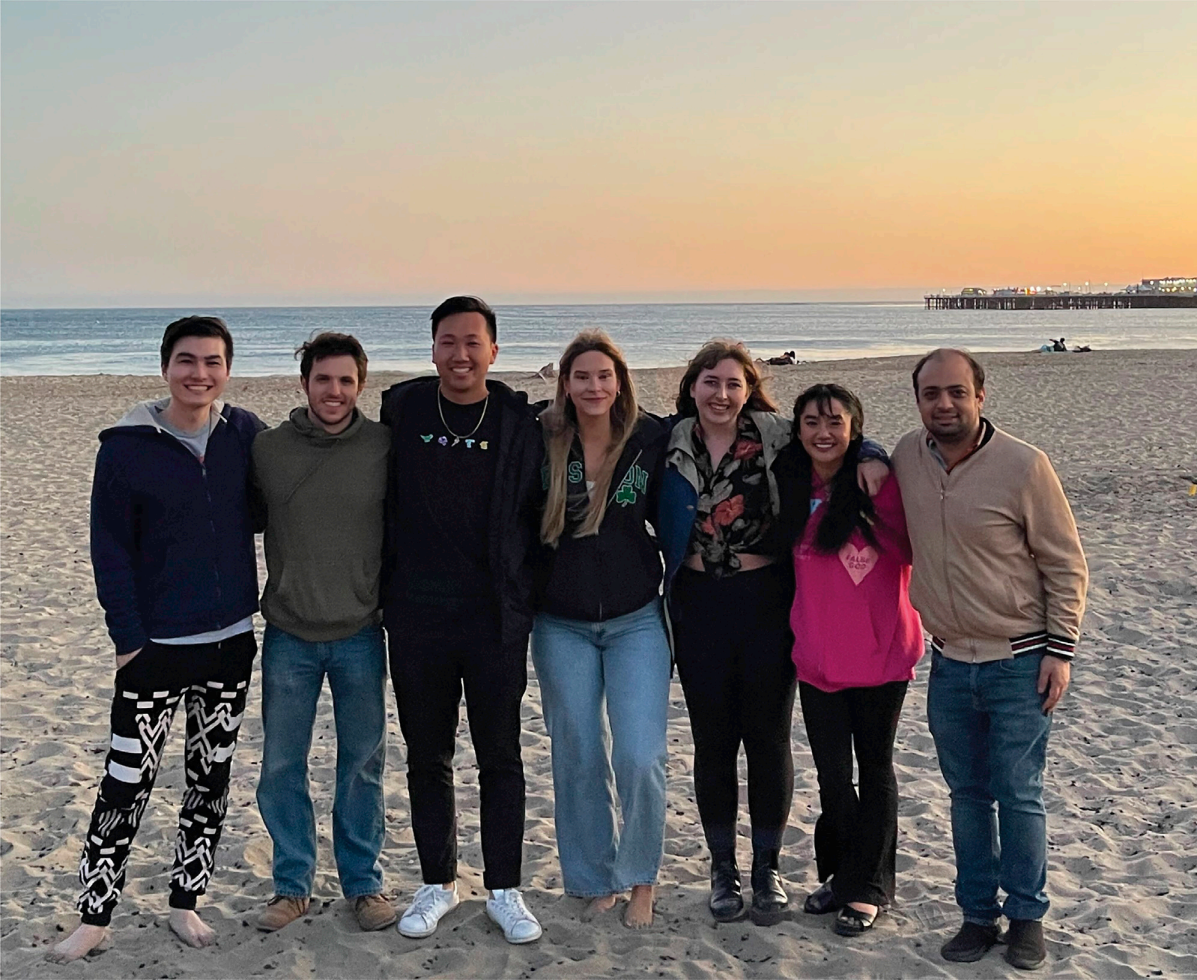
Training the next generation of neurodiplomats Mostajo-Radji leads an interdisciplinary team working on brain development and science education.

In the academic world, I believe the problem is different in nature. Although it is true that academia is the place where interdisciplinary research takes place, I feel that there is little space for individual people to be interdisciplinary. We often value the expert on a narrow topic over the people who want to merge several fields. For example, when I was wrapping up my postdoctoral training, I had meetings with my Ph.D. and postdoctoral advisors to discuss what was next for me. They both asked me to describe what would be the ideal position for me, to which I said: "I would like to have a lab that studies cortical development and education, ideally in a department where I can brainstorm projects with colleagues in completely different fields, such as engineering, and I would prefer if my teaching responsibilities were through a school of government." My advisors, Paola Arlotta and Alex Pollen are both amazing scientists and incredible human beings. They both know that if you meet me for 5 min, that "ideal situation" completely makes sense. But the harsh reality is that getting a department of Biology, engineering, politics, and education to come together in a job search is very unlikely. I am fortunate to have found at the UCSC Genomics Institute an independent position that allows me to follow both of my scientific passions, and I am preparing my course in science diplomacy as part of the UCSC Global Health Initiative while sharing the lab space with Mircea Teodorescu, a phenomenal electrical and computer engineer. There is something truly magical about having brain organoid cultures in the same room as 3D printers, robots, drones, and lasers. I want to mention that I am extremely thankful to David Haussler, who has helped me catalyze this role into a position in which I can thrive. One of the beauties of having an interdisciplinary role is collaborating with brilliant minds across divisions. I have been in this position for under a year. However, I already have strong collaborations and active projects with several teams, including Sofie Salama at Genomics, Sri Kurniawan at Computational Media, Marco Rolandi at Computer and Electrical Engineering, Vanessa Jönsson at Applied Mathematics, and Kristian Lopez-Vargas and Julian Martinez-Iriarte at Economics.

Yet, throughout my career, I have seen academia lose so many brilliant minds, because we cannot find a space for them to thrive. At the same time, I have seen several colleagues having to pick just one of their passions to follow. In my opinion, this is wrong and against the academic spirit to begin with. But the worst part is that this approach leaves behind the people who have had these unique life experiences, who are, by definition, underrepresented in the sciences. In a way, we create an academic oxymoron: we want to recruit new people with diverse backgrounds, but to do so, we put them through a rubric that does not account for those experiences.

### Education, governance, and societal impacts

How do you prepare your students/early career researchers for interdisciplinary research methods?

I am very lucky to have students from various fields working in topics related to neuroscience and science education. Both areas of research are equally part of my career and equally important. With the students in my group, we are constantly sharing and discussing literature in these fields, and I expect that they will collaborate with each other throughout their training. At the same time, I am part of the Brainengineers, which is a multi-institutional interdisciplinary group of neuroscientists, computer scientists, and engineers working together to standardize and scale human brain organoid research. Through this collaboration, we all get exposed to a variety of topics: from 3D printing of microscopes (Ly et al., 2021) and Internet-of-Things enabled technologies for the lab (Parks et al., 2021) to the evolution of neuronal subtypes (Schmitz et al., 2022). I believe these kinds of interactions are key to developing the next generation of scientists with an interdisciplinary mindset.

I also like to encourage my students to explore. This is the period in which they can discover what they really like. They should be going to conferences outside their field, and they should consider applying to internships in industry, governments, and nonprofits. This year, for example, I have one student that will be doing a summer internship in translational research at a different university and another interning at a large biotech company.

What are the challenges during publication in the field of Neurodiplomacy?

As in any new field, you must open the road as you walk it. This means that many times there are no journals where your paper would perfectly fit, and many times we have the discussion of whether it is easier to split the paper into two papers, which I tend to disagree on. For example, sometimes we write a paper describing a specific technology and how we believe this technology would advance an SDG. The first thing we have to do is to think who the final audience is, which in the best-case scenario would be scientists, as well as policy advisors and diplomats. Scientists are used to papers that explain every detail of the methodology and the results. On the other hand, policy briefs are usually succinct and focus more on the broader implications. Therefore, the second issue becomes writing the manuscript in a way that would be appreciated by all readers. Third is to find the journal that will want to work with us on the publication of this article. This part is probably the most difficult one as there are very few top journals that are truly interdisciplinary. Hence, the communication with the editors is key. For instance, one needs to think of a long list of potential reviewers who can accurately judge different aspects of the manuscript but also reviewers working at intersections of fields who would appreciate the interdisciplinary nature of the research.

### Future perspective

What are the next big questions and prospects in this field?

I believe the field will focus in five areas: neurorights, data governance, trade of neurotechnologies, education, and people-to-people exchange. We have already started to see major discussions happening at the highest levels in regards to neurorights and neuroethics. At the same time, we must keep in mind that neurotechnologies and treatment of neurological diseases is a $200 Billion market, which will only keep increasing. Therefore, guaranteeing equitable access to these technologies is a fundamental question to explore.

The COVID-19 pandemic has taught us that we can transition many roles to a remote environment. Yet, laboratory science, both in research and in education, is still falling behind. Indeed, during the pandemic, laboratory courses were disproportionately affected. Schools took several approaches, including using simulations and having students do simple experiments at home. Yet, none of these approaches is giving students a true discovery experience. But what if we could change that? What if we can get students to do high quality experiments at home in real time from anywhere in the world? This would truly allow for a revolution in science education, creating virtually unlimited opportunities for multinational collaborations. We are currently working on implementing such approaches (Baudin et al., 2021; Parks et al., 2021) and have completed our pilot studies.

What tips would you give to anyone considering undertaking such projects?

Neurodiplomacy is a new and exciting field with much space for growth, and the moment is now to ask the big questions and go after bold projects. At the same time, I would advise young scientists to spend the time picking mentors who will support them through an intrinsically interdisciplinary career.

## References

[bib1] Alegria M., Canino G., Shrout P.E., Woo M., Duan N., Vila D., Torres M., Chen C.N., Meng X.L. (2008). Prevalence of mental illness in immigrant and non-immigrant U.S. Latino groups. Am. J. Psychiatry.

[bib11] Barber K., Mostajo-Radji M.A. (2020). Youth Networks’ Advances Toward the Sustainable Development Goals During the COVID-19 Pandemic. Front. Sociol..

[bib2] Baudin P.V., Ly V.T., Pansodtee P., Jung E.A., Currie R., Hoffman R., Willsey H.R., Pollen A.A., Nowakowski T.J., Haussler D. (2021). Low cost cloud based remote microscopy for biological sciences. Internet Things.

[bib3] Carosso G.A., Ferreira L.M.R., Mostajo-Radji M.A. (2019). Developing brains, developing nations: can scientists be effective nonstate diplomats?. Front. Educ..

[bib4] Carosso G.A., Ferreira L.M.R., Mostajo-Radji M.A. (2019). Scientists as nonstate actors of public diplomacy. Nat. Hum. Behav..

[bib5] Ferreira L.M.R., Carosso G.A., Montellano Duran N., Bohorquez-Massud S.V., Vaca-Diez G., Rivera-Betancourt L.I., Rodriguez Y., Ordonez D.G., Alatriste-Gonzalez D.K., Vacaflores A. (2019). Effective participatory science education in a diverse Latin American population. Palgrave. Commun..

[bib6] Insel T.R., Landis S.C., Collins F.S. (2013). The NIH BRAIN initiative. Science.

[bib7] Ly V.T., Baudin P.V., Pansodtee P., Jung E.A., Voitiuk K., Rosen Y.M., Willsey H.R., Mantalas G.L., Seiler S.T., Selberg J.A. (2021). Picroscope: low-cost system for simultaneous longitudinal biological imaging. Commun. Biol..

[bib8] Mostajo-Radji M.A. (2021). Pseudoscience in the times of crisis: how and why chlorine dioxide consumption became popular in Latin America during the COVID-19 pandemic. Front. Polit. Sci..

[bib9] Parks D.E., Voitiuk K., Geng J., Elliott M.A.T., Keefe M.G., Jung E.A., Robbins A., Baudin P.V., Ly V.T., Hawthorne N. (2021). Internet of things architecture for cellular biology. biorxiv.

[bib10] Schmitz M.T., Sandoval K., Chen C.P., Mostajo-Radji M.A., Seeley W.W., Nowakowski T.J., Ye C.J., Paredes M.F., Pollen A.A. (2022). The development and evolution of inhibitory neurons in primate cerebrum. Nature.

